# The retinal pigment epithelium of the eye regulates the development of scleral cartilage

**DOI:** 10.1016/j.ydbio.2010.08.006

**Published:** 2010-11-01

**Authors:** H. Thompson, J.S. Griffiths, G. Jeffery, I.M. McGonnell

**Affiliations:** a Department of Veterinary Basic Science, Royal Veterinary College, London, NW1 0TU, UK; b Institute of Ophthalmology, University College London, London, EC1V 9EL, UK

**Keywords:** Eye, Development, Sclera, Cartilage, RPE, Neural crest

## Abstract

The majority of vertebrate species have a layer of hyaline cartilage within the fibrous sclera giving an extra degree of support to the eyeball. In chicks, this is seen as a cuplike structure throughout the scleral layer. However, the mechanisms that control the development of scleral cartilage are largely unknown.

Here we have studied the phases of scleral cartilage development and characterised expression profiles of genes activated during the cartilage differentiation programme. *CART1* and *SOX9*, the earliest markers of pre-committed cartilage, are expressed in the mesenchyme surrounding the optic cup. Later *AGGRECAN,* a matrix protein expressed during chondrocyte differentiation, is also expressed. The expression of these genes is lost following early removal of the optic cup, suggesting a role for this tissue in inducing scleral cartilage. By grafting young retinal pigment epithelium (RPE) and retina into cranial mesenchyme *in vivo*, it was found that RPE alone has the ability to induce cartilage formation.

There are some exceptions within the vertebrates where scleral cartilage is not present; one such example is the placental mammals. However, we found that the cartilage differentiation pathway is initiated in mice as seen by the expression of *Cart1* and *Sox9*, but expression of the later cartilage marker *Aggrecan* is weak. Furthermore, cartilage forms in mouse peri-ocular mesenchyme micromass culture. This suggests that the process halts *in vivo* before full differentiation into cartilage, but that murine scleral mesenchyme has retained the potential to make cartilage *in vitro*.

RA, Wnts and Bmps have been linked to the cartilage development process and are expressed within the developing RPE. We find that RA may have a role in early scleral cartilage development but is not likely to be the main factor involved.

These data reveal the course of scleral cartilage formation and highlight the key role that the optic cup plays in this process. The driving element within the optic cup is almost certainly the retinal pigmented epithelium.

## Introduction

**Fig. 1:**
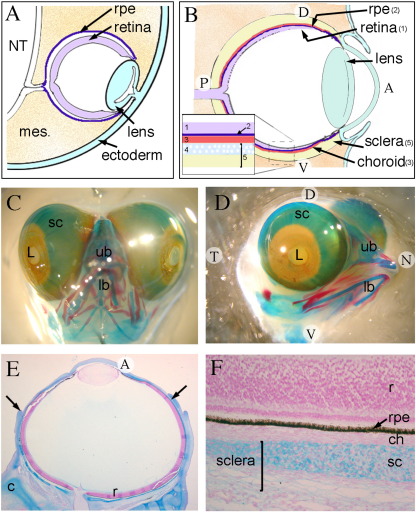
Cartilage is found within the scleral layer of the eye. (A) Schematic showing the structure of the eye early in development, HH stages 13–27 (E2–E5) composed of the CNS-derived layers the retinal pigment epithelium (RPE) and retina surrounded by the neural crest-derived mesenchyme (mes). (B) Schematic showing the structure of the mature eye with all its layers. (C–D) Alcian blue and alizarin red staining of whole-mount HH stage 36 (E10) chick embryos showing scleral cartilage (sc) surrounding the eyes, except in the most anterior region of the eye near the lens (L). (E–F) Alcian blue and nuclear fast red staining on sections, of HH stage 36 (E10) chick showing scleral cartilage (arrows in E) within the sclera layer, adjacent to the choroid (ch) and at a short distance from the RPE (F). NT, neural tube; L, lens; ub, upper beak; lb, lower beak; r, retina; c, cartilage; 1—retina; 2—RPE; 3—choroid; 4—scleral cartilage; 5—sclera.

The vertebrate eye develops as bilateral diencephalic outgrowths, which protrude into surrounding peri-ocular mesenchyme. These contact the surface ectoderm before invaginating to form optic cups composed of inner and outer neural ectoderm layers—the neural retina and retinal pigment epithelium (RPE) respectively (Fig. 1A). With development, these layers divide and differentiate into their mature cells types, while the surrounding mesenchyme condenses and develops into the choroid and scleral layers (Figs. 1A, B). The choroid layer consists of a vascular network supplying the outer retina. It also contains neural crest-derived melanocytes that pigment just prior to birth (Lopes et al., 2007). The sclera, which provides the eye with structural integrity is derived solely from neural crest (Le Lievre and Le Douarin, 1975; Johnston et al., 1979; Gage et al., 2005) and is a dense fibrous layer continuous with the cornea. The eye therefore develops from a number of distinct tissues from different developmental origins involving tightly controlled reciprocal signalling to coordinate growth and development.

In the majority of vertebrate species the sclera contains cartilage and in some cases also bone, which give an extra degree of support to the eyeball (Caprette et al., 2004; Franz-Odendaal and Vickaryous, 2006). Commonly this is a hyaline cartilage layer forming a cuplike structure within the sclera. In many teleosts, reptiles and birds the sclera also forms ossicles—bony plates found in a ring surrounding the anterior region of the eye (Franz-Odendaal and Vickaryous, 2006; Franz-Odendaal, 2008). Although most vertebrates develop a cartilage layer within the sclera, placental mammals, snakes, hagfish and lamprey do not (Caprette et al., 2004). Furthermore, in some amphibians, scleral cartilage is absent at the tadpole stage but present after metamorphosis (Franz-Odendaal and Hall, 2006). The mechanisms that control the development of the scleral cartilage are largely unknown and equally the mechanisms that prevent its formation in the mammals, agnatha and snakes are not understood.

Cartilage development has been extensively studied, most notably in the developing limb buds (reviewed by Shimizu et al., 2007). Here it has been shown that chondrogenesis occurs in stages beginning with mesenchymal cell condensation and proliferation followed by differentiation of chondrocytes and secretion of the cartilage matrix. The first phase is associated with the expression of cell adhesion molecules and the transcription factors *Sox9* and *Cart1* (A*lx1*) (Zhao et al., 1994; Wright et al., 1995). The secondary phases of chondrocyte differentiation and maturation correlates with expression of the transcription factors *Sox5* and *Sox6* (Lefebvre et al., 1998) and the extracellular matrix proteins collagen type II (Swalla et al., 1988) and aggrecan. In the majority of embryonic cartilage elements a process of hypertrophy and replacement by bone then takes place. However, there are isolated examples of where this does not occur and cartilage persists into adulthood, including the scleral cartilage. This gene expression sequence and the time window over which cartilage develops are similar in all mesodermal skeletal elements. The period over which neural crest-derived craniofacial cartilage differentiates is much longer however and few studies draw attention to this key difference. This delay in differentiation may reflect the need to coordinate growth with adjacent neural tissue and implies complex signalling between neural and skeletal structures.

The induction of cartilage development in both mesodermal and neural crest tissues is known to involve epithelial–mesenchymal interactions. This is particularly well characterised in the limb (mesodermal) (Hall, 1981) and in the mandible (neural crest derived) (Hall, 1981; Haworth et al., 2007), where surface ectoderm induces cartilage. More recently, the endoderm has been implicated in inducing pharyngeal cartilage development (Couly et al., 2002; Walshe and Mason, 2003; Crump et al., 2004). However, the developing scleral cartilage is not located close to either a surface ectoderm or an endoderm. Several *in vitro* studies in the 1970s suggested that the neuro-ectodermally derived RPE may induce cartilage (Newsome, 1972; Stewart and McCallion, 1975); however, they lacked specific markers to prove this.

Here, we will examine the sequence of scleral cartilage development in chick using histology and in situ hybridisation against various cartilage markers. We will also examine the affect of enucleation on scleral cartilage formation, examine which tissues in the optic cup can induce cartilage formation *in vivo* and look for the secreted molecules that may be involved. Finally, to determine if mammalian sclera initiates cartilage development we will examine expression of cartilage markers in mouse embryos and the ability of the mouse scleral mesenchyme to make cartilage.

## Methods

### Animals

Fertile white leghorn chicken eggs (Henry Stewart and Co. Ltd, Lincolnshire, UK) and Japanese Coturnix quail eggs (www.quails-in-essex.co.uk) were incubated at 37 °C, staged according to Hamburger and Hamilton (1951) or Ainsworth et al. (2010) and the extra-embryonic membranes removed before fixing in 4% PFA in PBS.

CD1 mice were obtained from in-house breeding or a gift from A. Basson (Craniofacial department, KCL). C57 (bl) were ordered from Charles River UK Ltd, Margate. Noon on the day the vaginal plug was found was considered embryonic day 0.5 (E0.5). Pregnant mothers were killed by cervical dislocation and the embryos removed and fixed in 4% PFA in PBS. All conditions and all experimental procedures were in accordance with the UK Animals (Scientific Procedures) Act 1986 and associated guidelines.

### Enucleations

A hole was made in the blunt end of an embryonic day 2 (E2) egg and 2 ml of albumin removed. Sellotape was stuck to the upper surface and a “window” (approximately 1 × 2 cm) was cut to reveal the embryo and an opening made in the chorion and amnion directly above the eye. Embryos at HH stage 12–14 were selected for operation and a flame sharpened tungsten needle was used to carefully cut around the optic cup, sever the optic stalk and remove the optic cup from the egg. The eggs were sealed with Sellotape and re-incubated before collecting at various points up to HH stage 39 (E13) and fixing in 4% PFA in PBS overnight.

### Grafting

HH stage 19 (E3) chick or Q stage 19 (E3) quail embryos were enucleated and the eye transferred to HBSS (without calcium and magnesium, Sigma, Dorset, UK). Using a needle and fine forceps the RPE was peeled away from the retina. Each tissue was then sliced into 3–4 pieces for grafting. For the host, HH stage 12–14 (E2) chick embryo was enucleated and the graft was pushed into the cavity left a by optic cup removal. In other experiments, a HH stage 18-19 (E3) chick egg was opened and a small incision was made in the ectoderm temporal to the eye using a needle. The graft was pushed into the ectoderm incision using needles. The eggs were sealed with Sellotape and re-incubated for up to 7 days (HH stage 36) before collecting and fixing in 4% PFA in PBS overnight.

For mouse grafting, E10.5 or E11.5 embryos were harvested and transferred to L15 media (Sigma) on ice before preparing the tissue for grafting as above.

### RA bead implantation

AG1X-2 beads were soaked in 0.1 mg/ml or 1 mg/ml retinoic acid (RA; Sigma) dissolved in dimethylsulfoxide (DMSO) for 3 h, rinsed in PBS then in L-15 media. Embryos were enucleated as above and a RA bead was pushed into the cavity left by the removed optic cup. The eggs were sealed with Sellotape and re-incubated for 2 days (HH stages 21–23) before collecting and fixing in 4% PFA in PBS overnight. Control embryos were treated with DMSO-soaked beads.

### Micromass culture

Embryonic day 12.5 embryos were collected in HBSS media (calcium- and magnesium-free; Sigma) and the head was trimmed to leave only the area around the eye before incubating in 2% trypsin for 30 minutes at 4 °C. Following this the tissue was washed in L15 (Sigma) with 10% FCS (Sigma) and the ectoderm and optic cup tissue was carefully peeled away from the mesenchyme leaving a small band of peri-ocular mesenchyme. This was then cultured as a micromass (Cottrill et al., 1987) at 2 × 10^7^ cells per ml, for 4 days in Hams F12/DMEM media (1:1) with 10% FCS and penicillin/streptomycin. The cultures where then fixed in 4% PFA, rinsed in 3% acetic acid and stained with 1% Alcian blue/3% acetic acid (1:1) to label cartilage nodules.

### Alcian blue staining of cartilage

Embryonic heads were dehydrated in 95% ethanol for 2 days, soaked in acetone for 2 h before staining with 0.3% Alcian blue (VWR, Poole, UK) in 70% ethanol (with or without a 0.1% alizarin red (Sigma) in 95% ethanol) in acetic acid and 70% ethanol at a ratio of 1:1:1:17 (freshly made), for 5 days at 37 °C. Embryos were rinsed in distilled water, cleared in a decreasing concentration of 1% potassium hydroxide in glycerol and stored in 100% glycerol.

For staining of tissue sections, embryonic heads were embedded in paraffin and sectioned transversely at 5 μm, before dewaxing and rehydrating. Sections were then stained with a 1% aqueous Alcian blue in 1% aqueous acetic acid solution for 10 minutes, followed by 3 minutes in a nuclear fast red solution (Vector Labs, Peterborough, UK), then dehydrated, washed in xylene and mounted with DPX.

### Haematoxylin and eosin staining

Embryonic heads were fixed in 4% PFA, embedded in paraffin wax before sectioning transversely at 8–16 μm. Slides were dewaxed, rehydrated and stained with haematoxylin and eosin (H&E) before dehydrating and mounting in DPX.

### In situ hybridisation

Whole-mount in situ hybridisation was performed (Thompson et al., 2010) using digoxigenin-labelled riboprobes against chick *CART1*, *SOX9* (Healy et al., 1999), *AGGRECAN* (gift from Prof. Hurle, Chimal-Monroy et al., 2003), *AXIN2* (a gift from Prof Francis West) or *CRABP1* (Maden et al., 1992) or mouse *Cart1* (a gift from Prof. P. Sharpe), *Sox9* (a gift from Prof. P. Sharpe) or *Aggrecan* (gift from Dr. A Grigoriadis, Glumoff et al., 1994). Embryos were viewed as whole mounts or sectioned. For sectioning, heads were placed in 20% gelatine/PBS for 1 h at 55 °C, embedded, fixed overnight in ice-cold 4% PFA/PBS and sectioned transversely at 50 μm on a vibratome or cryo-embedded and sectioned at 25 μm on a cryostat and mounted using 90% glycerol PBS or stained with nuclear fast red (Vector Labs) for 1 minute before mounting in 90% glycerol PBS.

In situ hybridisation on 8–12 μm paraffin sections was performed in the same way as whole mount in situ but with the following alterations. Prior to hybridisation, an acetylation step was performed (0.25% acetic anhydride (Fluka)/0.1 M TEA-HCl) for 10 minutes. The hybridisation buffer consisted of 50% formamide, 1% Dextran sulphate, 10% Denhardt's, 250 μg/ml yeast RNA, 0.3 M NaCl, 20 mM Tris–HCl, 5 mM EDTA, 10 mM Na3PO4, 1% *N*-lauroylsarcosine sodium salt and slides were washed in 50% formamide, 2× SSC. Following in situ hybridisation the sections were counterstained with nuclear fast red for 1 minute and mounted in 90% glycerol/PBS.

Mouse section in situ hybridisation (Thompson et al., 2006) was performed on cryosections. Embryonic heads were sectioned horizontally at 30 μm. Digoxigenin-labelled riboprobes against mouse *Cart1*, *Sox9* or *Aggrecan* were used and sections were mounted in 90% glycerol/PBS.

### Immunohistochemistry and cell counts

Monoenucleated embryonic heads were cryo-embedded and sectioned transversely at a thickness of 14 μm. The section containing the optic disk was used as a marker and was taken along with 7 consecutive sections. These were incubated with rabbit IgG anti-phospho-histone H3 (Ser 10) (Upstate Biotechnology, Lake Placid, NY; 1:100) followed by goat anti-rabbit HRP secondary antibody (Invitrogen; 1:200). The number of mitotic cells was counted in a 150-μm^2^ box from each section in a total of 5 animals. On the non-enucleated control side of the embryo, the box was positioned temporal to the eye abutting the RPE and ectoderm so as to contain only mesenchyme cells. A box was positioned on the enucleated side of the section in an equivalent position. Statistical analysis was performed using the paired *t*-test.

### Immunostaining

Embryonic heads were fixed in 4% PFA, embedded in paraffin wax before sectioning transversely at 8–16 μm. Slides were dewaxed, rehydrated and treated with 0.03% hydrogen peroxide for 10 minutes. Antigen retrieval was performed in boiling 0.04% citric acid solution pH6 (Sigma) for 10 minutes. Sections were then digested with 0.25 units/ml chondroitinase ABC (Sigma) and 1.45 units/ml hyaluronidase (Sigma-Aldrich) at 37 °C for 30 minutes before overnight incubation in mouse anti-QCPN (1:1; DSHB, Iowa City, USA), rabbit polyclonal anti-collagen type II (1:15; Novocastra/Leica, Newcastle, UK), rabbit polyclonal anti-Phospho-Smad1/5/8 (1:250; without enzyme treatment; Cell Signalling/New England Biolabs, Hitchin, UK) or rabbit polyclonal anti-Sox9 (1:100; Millipore, UK). The sections were washed and incubated in goat anti-rabbit-488 (Molecular probes/Invitrogen, Paisley, UK), goat anti-mouse-546 (Molecular probes) or goat anti-mouse-HRP. The sections were washed and either developed with DAB or mounted in VectorMount with DAPI (VectorLabs) and imaged under a Leica DM4000B fluorescent microscope.

## Results

### Identification of scleral cartilage in ocular mesenchyme

Alcian blue (cartilage) and alizarin red (bone) staining of whole embryonic chick heads was performed to visualise the scleral cartilage cup surrounding the eyes at HH stage 36 (E10) (Figs. 1C, D). At this stage the eyes are relatively large and protrude out of the skull. The scleral cartilage can clearly be seen, covering the majority of the eye. Alcian blue staining is not present in the anterior most part of the eye, namely the cornea over the lens and iris. A number of other cranial cartilage elements are beginning to form and ossification of bone has begun with those most developed found in the beak. At this stage, bone is not seen within the chick sclera.

To determine the exact location of the scleral cartilage in relation to the other components of the eye, HH stage 36 chick heads were sectioned and the cartilage stained with Alcian blue and counterstained with nuclear fast red. At this stage, cartilage encircles the eye surrounding the central and peripheral regions of the retina (Fig. 1E) at a slight distance from the RPE, with the blood vessels and cells of the choroid between those two layers (Fig. 1F). This cartilage structure forms a cuplike shape with a hole centrally, through which the optic nerve passes and is separated from the RPE by a thin layer of choroid tissue.

### Phases of scleral cartilage development

**Fig. 2:**
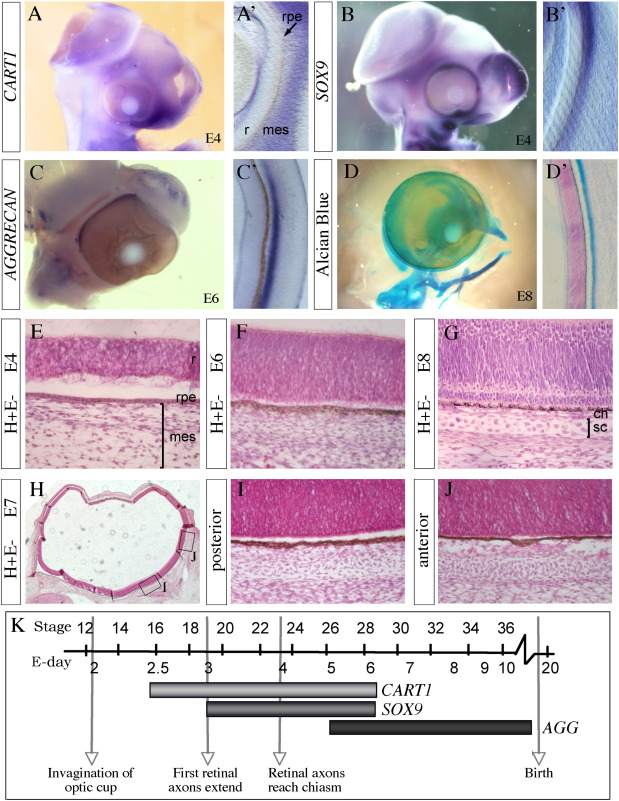
The development of chicken scleral cartilage. (A–C) In situ hybridisation showing expression of *CART1* (A, A′), *SOX9* (B, B′) and *AGGRECAN* (C, C′) genes in chick embryos, in whole-mount (A, B, C) and in section (A′, B′, C′). Images are of the nasal (A′, B′) or dorsal (C′) region of eye. *CART1* is expressed as a thick band around the optic cup (A) in the mesenchyme adjacent to the RPE (arrow; A′) with stronger expression seen nasally seen here at HH stage 23 (E4). Expression of *SOX9* is seen as a narrow band surrounding the optic cup seen here at HH stage 24 (E4; B, B′). *AGGRECAN* is expressed strongly as a thin band around the chick optic cup (C), at a slight distance from the RPE (C′) seen here at HH stages 28and 29 (E6). Alcian blue staining of HH stage 34 (E8) chick in whole mount (D) and in section (D′), showing a thin band of cartilage surrounding the optic cup at a short distance from the RPE. (E–G) H&E staining on paraffin sections at HH stages 22–24 (E4; E), HH stages 28 and 29 (E6; F) and HH stage 34 (E8; G). The loose mesenchyme at E4 is starting to condense close to the RPE (E), by E6 the mesenchyme is highly packed but no definitive chondrocytes can be seen (F). At E8 (G), a choroid layer (ch) is seen adjacent to the RPE and definitive cartilage (sc) is seen adjacent to the choroid. (H–J) H&E staining on paraffin sections at HH stages 30 and 31 (E7) showing the differing stages of cartilage development around the eye. Cartilage development is less advanced in the posterior (I) than anterior regions (J). (K) Diagram illustrating when cartilage markers are expressed in relation to retina development. *CART1* is expressed from HH stages 15–29 (E2.5 < E6), *SOX9* is seen from HH stages 18–29 (E3 < E6) and *AGGRECAN* is seen from HH stage 26 (E5) onwards. Mes, mesenchyme; *AGG, AGGRECAN*.

To characterise the development of the scleral cartilage we analysed a panel of genes that mark different stages of cartilage differentiation. The earliest marker present in the ocular mesenchyme is C*ART1*, which is a very early marker of pre-committed cartilage. It is expressed from HH stage 16 (E2.5) and by HH stage 23 (E4) is seen in a broad band around the optic cup with stronger expression nasally (Fig. 2A). Sections indicate expression is in the mesenchyme adjacent to the RPE (Fig. 2A′). This expression persists up to HH stage 29 (E6) (Fig. 2K). *SOX9*, also a marker of pre-committed cartilage, is clearly detected completely surrounding the optic cup. Expression is seen in a narrow band in the ocular mesenchyme at HH stage 23 (E4; Figs. 2B, B′) following initiation at HH stage 18 and continues until approximately E6 (Fig. 2K). *AGGRECAN* is a matrix protein that is first expressed as chondrocytes differentiate; it is expressed in the ocular mesenchyme and is initially seen in patches from HH stage 25 (E5) and more robustly from HH stage 28 (E6) (Figs. 2C, K). In contrast to the early cartilage markers, its expression is restricted to a tight band at a slight distance from the RPE (Fig. 2C′). The tissue between the RPE and the *AGGRECAN* expressing cartilage is the developing choroid. The *AGGRECAN* expression pattern is similar to the Alcian blue staining seen at HH stage 34 (E8, Figs. 2D, D′), which shows a thin band of staining at a slight distance from the RPE. *AGGRECAN* expression and Alcian blue staining differentiates the developing scleral cartilage from the non-cartilage sclera (fibrous sclera) and choroid. From the earliest gene expression up to definitive cartilage formation and matrix production, this process comprises of around 5.5 days.

To correlate these expression patterns with cellular events, we examined H&E stained sections of the ocular territory. Early cartilage marker expression patterns (*CART1*, *SOX9*) coincide with condensation of mesenchyme adjacent to the RPE, which can be seen robustly at HH stages 23-24 (E4) (Fig. 2E). By HH stage 29 (E6), mesenchyme at a distance from the RPE has also condensed and even though *AGGRECAN* is marking future scleral cartilage clearly, these cells cannot be distinguished from choroid or fibrous sclera with H&E (Fig. 2F). By HH stage 34 (E8) a definitive cartilage is present with chondrocytes surrounded by matrix (Fig. 2G). At this stage there is an obvious choroid that is highly vascularised but not yet pigmented. Interestingly, the scleral cartilage in the posterior (central) eye region (Figs. 2H, I) is less differentiated compared to that found more peripherally in the eye (anterior regions; Figs. 2H, J).

### CNS-derived tissues are necessary for the development of scleral cartilage

**Fig. 3:**
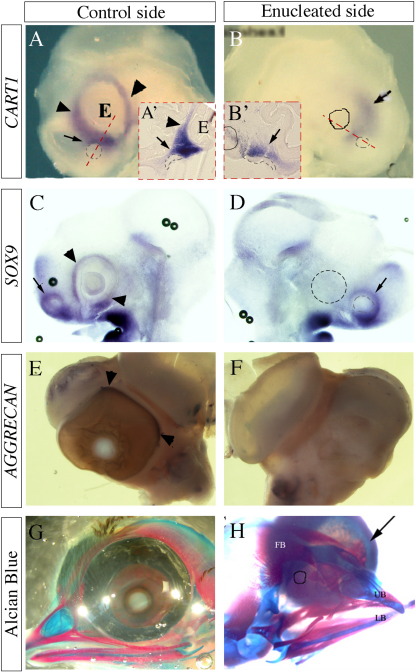
Enucleation leads to loss of cartilage markers in adjacent mesenchyme. Sectioned (A′, B′) and whole-mount embryos (A, B, C, D, E, F, G, H) of the control (left) side of the embryo (A, A′, C, E, G) compared to the enucleated (right) side of the embryo (B, B′, D, F, H) showing expression of *CART1* (A, A′, B, B′), and *SOX9* (C,D), both at HH stage 18/9 (E3) *AGGRECAN* at HH stage 29 (E6; E, F) and staining with Alcian blue at HH stage 39 (E13; G, H). Enucleations were performed at HH stage 13/4 (E2). *CART1, SOX9* and *AGGRECAN* are expressed around the eye on the control side of the embryos (arrow heads in A, A′, C, E). Expressions of *CART1, SOX9* and *AGGRECAN* are lost from the peri-ocular region following enucleation (B, D, F). *CART1* and *SOX9* are expressed (arrows) around the nasal placode both on the control and enucleated sides (dotted grey line; A, A′, B, B′ C, D). Scleral cartilage can be seen encircling the eye on the control side of the embryo when stained with Alcian blue (G); however, no staining is seen in the place where the eye would be on the enucleated side. Cartilage can be clearly seen surrounding the contralateral control eye (arrow) (H). Circles in B, D and H indicate the position where the eye would have been. E; eye.

To investigate the role of the CNS-derived ocular tissue in scleral cartilage development *in vivo*, the right optic cup was removed from HH stage 12–14 (E2) embryos. Embryos were re-incubated for 12 h to 4 days before analysing cartilage gene expression or staining with Alcian blue to visualise the cartilage matrix. The early cartilage markers *CART1* and *SOX9* are expressed in the mesenchyme surrounding the optic cup (Figs. 3A, C) as described above. Following enucleation, this expression is lost in the peri-ocular mesenchyme (HH stage 19 (E3) Figs. 3B, D). Analysis at HH stages 15-16 indicated that expression of *CART1* was not initiated following enucleation (data not shown). However, expression of both genes is still seen adjacent to the nasal placode (Figs. 3A′ and B′), which is known to be required for nasal/upper jaw skeletal development (Szabo-Rogers et al., 2009). To confirm that definitive cartilage does not form, *AGGRECAN* expression and Alcian blue staining were assessed. *AGGRECAN* expression is absent from the enucleated side of the embryo, compared to the control (compare Fig. 3E with Fig. 3F). *AGGRECAN* expression is present within the otic capsule on both control and enucleated sides. Alcian blue staining was also absent in the region where the eye would have developed (compare Figs. 3G and H). This demonstrates the presence of the CNS-derived epithelia is required for scleral cartilage to form *in vivo*.

**Fig. 4:**
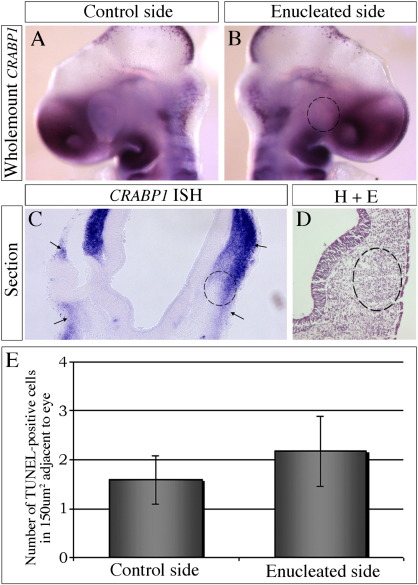
Neural crest cells are still present following enucleation. In situ hybridisation of *CRABP1* in whole mount (A, B) or in cryosection (C) showing neural crest cells surrounding the control (left) optic cup (A, C), and in the area where the optic cup would have been (circle) in the enucleated (right) side of the embryo (B, C), at HH stage 19 (E3). Arrows in C indicate neural crest cells. This shows neural crest cells were not removed during the enucleation and can be found surrounding the eye at E3. (D) H + E staining of an HH stage 19 (E3) embryo enucleated at HH stage 13 (E2) showing cells filling the region of the head where the eye had been removed. (E) A graph showing the number of TUNEL-positive cells in a 150-μm^2^ area temporal and adjacent to the optic cup and in the corresponding region on the enucleated side of the embryo. The embryo was enucleated at HH stage 13 (E2) and counts were performed at HH stage 19 (E3). There is no significant difference in the number of TUNEL-positive cells (*p* < 0.05).

Loss of scleral cartilage within orbital mesenchyme could be due to removal or damage of neural crest cells during enucleation or inability of these cells to migrate into the correct region. To determine if this was the case, in situ hybridisation was performed using the neural crest marker *CRABP1*, which labels a large number of neural crest cells within the head of the HH stage 19 (E3) embryo (Figs. 4A, C). *CRABP1* expression was not changed in the peri-ocular mesenchyme after enucleation, confirming that neural crest cells were present in the head in the region where the optic cup was removed (Figs. 4B, C). H&E staining shows mesenchymal cells have filled the region where the optic cup was removed and appear to be developing in the same way as the surrounding mesenchyme (Fig. 4D). TUNEL analysis was performed comparing the number of peri-ocular mesenchymal cells dying in the enucleated side of the embryo with the equivalent region in the contralateral control. An average of 1.6 TUNEL-positive cells was found in the mesenchyme on the control side of the embryos compared to 2.2 TUNEL-positive cells in the mesenchyme on the enucleated side (Fig. 4E). Even though there is a slight increase following enucleation, this was not significantly different (paired *t*-test; *p* < 0.05). Therefore, it is loss of the CNS-derived layers of the eye not loss of the ocular mesenchyme that results in lack of scleral cartilage development.

### The RPE induces cartilage development

**Fig. 5:**
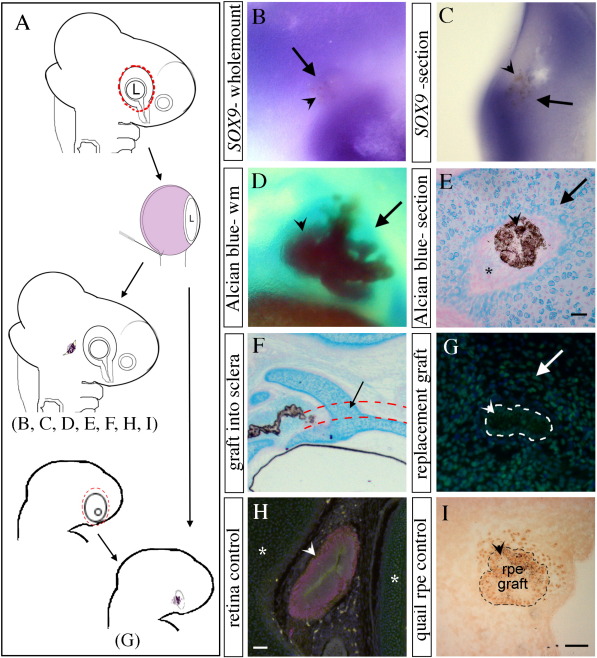
RPE induces cartilage in cranial neural crest and presumptive fibrous sclera. (A) Schematic illustrating how the grafting experiments were performed. First the optic cup of an HH stage 19 (E3) chick or Q stage 19 (E3) quail embryo was removed, then the RPE or retina was dissected away from the other ocular tissues and grafted into the head of an HH stage 19 (E3) chick embryo or into the space left by the removed optic cup in an HH stage 13 (E2) chick embryo. (B–C) In situ hybridisation using a probe against *SOX9***,** seen as whole-mount (B) or in section (C) on HH stages 22–24 (E4) embryos following grafting at HH stage 19 (E3). Expression of *SOX9* (arrow) is seen around the grafted pigmented RPE tissue (arrow head). (D–E) Alcian blue staining of cartilage seen in whole-mount HH stage 34 (E8) embryos (D) or in section with nuclear fast red staining at HH stage 36 (E10) (E), following grafting at HH stage 19 (E3). Alcian blue staining (arrow) is seen around the ectopic RPE graft (arrow head). There is a gap between the cartilage and the graft (*) equivalent to the choroid layer in the normal eye. (F) Alcian blue staining in section at HH stage 36 (E10) with an RPE graft positioned close to the eye showing the ectopic induction of cartilage (arrow) within tissue that would normally form fibrous sclera (bounded by red dashed line) and in mesenchyme further away from the eye. (G) Immunostaining against SOX9 (green) with DAPI labelling the nuclei (blue) of sectioned HH stage 28 (E6) showing an RPE graft (arrow head) that was placed in the hole left by the removed optic cup at HH stages 12–14 (E2). This shows a rescue of SOX9 expression. (H) Immunostaining against QCPN labels the quail retina graft (red) and collagen II labels cartilage (green) with DAPI labelling nuclei (blue). The retina does not induce cartilage. (I) Grafting with Q stage 19 (E3) quail RPE into HH stage 19 (E3) chick and immunostaining with QCPN shows only a small number of mesenchyme cells are transferred along with the RPE graft. These are found very close to the graft. Scale: 250 μm (E), 500 μm (H) and 200 μm (I).

Previous *in vitro* studies suggested that RPE might induce the formation of cartilage from cranial neural crest (Newsome, 1972; Stewart and McCallion, 1975; Smith and Thorogood, 1983). To demonstrate that this occurs *in vivo*, RPE from HH stages 18-19 (E3) embryos was dissected away from neural retina and grafted into equivalent stage cranial mesenchyme temporal to the eye, in a region where cartilage does not normally form (Fig. 5A). To determine if the RPE graft has the ability to induce the earliest cartilage markers, embryos were collected at HH stages 22–24. Of the 22 chicks grafted, 10 survived of which 8 retained the graft (36% success rate). In 6 of these, *SOX9* expression was analysed using in situ hybridisation. At this stage the RPE has become pigmented and can be clearly distinguished (arrow head in Figs. 5B, C) within the mesenchyme just under the surface of the ectoderm (Fig. 5C). In 3/6 embryos analysed, the graft was surrounded by *SOX9* expressing cells, indicating that RPE has the ability to initiate the cartilage development pathway in this mesenchyme.

**Fig. 6:**
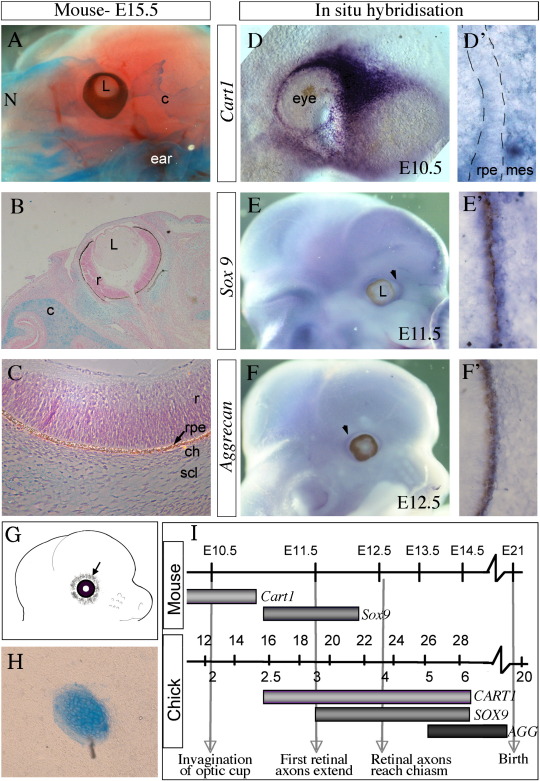
Mouse peri-ocular mesenchyme expresses early cartilage markers and has the ability to produce cartilage *in vitro*, but no cartilage forms *in vivo*. Whole-mount Alcian blue and alizarin red stain (A) and sectioned Alcian blue and nuclear fast red stain (B–C) of E15.5 mouse embryo showing absence of cartilage surrounding the eye or near the RPE. However, other cranial cartilages have formed by this stage (c). (D–F) In situ hybridisation showing expression of C*art1* at E10.5 (D, D′), *Sox9* at E11.5 (E, E′) and *Aggrecan* at E12.5 (F, F′) in mouse in whole-mount (D, E, F) and in section (D′, E′, F′). *Cart1* is expressed around the optic cup with stronger expression nasally (D, D′) at E10.5 (I), *Sox9* is expressed around the optic cup adjacent to the RPE (E′) at E11.5 (I) the expression is weak and cannot be seen in whole-mount (E). *Aggrecan* is not expressed in the mouse adjacent to the RPE at any significant level (F, F′, I). *Sox9* and *Aggrecan* expression can be seen in the eyelids at a slight distance from the eye (arrow heads in E, F). (G) Schematic illustrating the location of the peri-ocular mesenchyme from which the neural crest cells were taken for micromass culture. (H) An Alcian blue stained micromass culture of mouse peri-ocular mesenchyme, showing an example of a cartilage nodule. (I) Diagram illustrating when cartilage markers are expressed. C, cartilage; scl, sclera; mes, mesenchyme.

To assess whether RPE grafts could induce the formation of mature cartilage *in vivo*, embryos were incubated up to HH stage 34 (E8) and stained with Alcian blue. Of 72 embryos grafted, 14 survived of which 6 retained grafts (8% success rate). A large pigmented graft (arrowhead in Fig. 5D) could be seen temporal to the eye, and in all embryos this was surrounded by strong Alcian blue staining (*n* = 6/6). In order to see definitive cartilage cells, grafted embryos were also left until HH stage 36 (E10) and sectioned (12 grafted, 3 survived, 3 with grafts) before staining with Alcian blue or immunostaining against collagen II (data not shown). In all embryos examined (*n* = 3/3), the RPE graft was surrounded by a large mass of ectopic cartilage (Fig. 5E). Interestingly, cartilage cells did not abut the graft, but formed at a slight distance from it, reminiscent of the architecture of the ocular mesenchyme, where the choroid layer forms between RPE and cartilage (Fig. 1F). Of those grafted embryos where *SOX9* was not induced (*n* = 3), one had the graft in a position next to the neural tube where there is little mesenchyme. The remaining 2 embryos may have not been in place for sufficient time to induce *SOX9* expression, as all grafts left for longer incubation times eventually formed ectopic cartilage (Figs. 5D, E; 6 at HH stage 34 and 3 at HH stage 36). In these experiments, most of the RPE grafts were found at a slight distance from the eye. A few of them, however, were found within the developing fibrous sclera. These grafts were surrounded by ectopic cartilage (Fig. 5F) showing RPE has the ability to transform fibrous sclera into mature scleral cartilage.

To determine whether RPE has the ability to rescue the cartilage development pathway lost following optic cup removal, HH stages 18-19 (E3) RPE was grafted into the cavity left by optic cup removal in an HH stages 12–14 (E2). Embryos were collected after 3 days incubation (8 grafted, 8 survived, 5 with grafts) and cartilage development analysed using the anti-SOX9 antibody, which showed induction in cells completely surrounding the graft (Fig. 5G).

In contrast to the RPE's ability to induce cartilage, grafts of the neural retina do not induce cartilage (Fig. 5H; *n* = 3/3). Quail neural retina was grafted in the same way as the RPE and an anti-QCPN antibody used to identify transplanted tissue in the head mesenchyme and a collagen type II antibody used to detect definitive cartilage. Alcian blue staining could not be used to detect cartilage on slides immunostained for QPCN, as the enzymatic treatment required for this antibody digests the extracellular matrix that binds Alcian blue. Thus, it is the RPE and not neural retina that has the ability to induce cartilage in mesenchyme.

To confirm that RPE grafts do not transfer significant amounts of mesenchyme, quail RPE was grafted and anti-QCPN antibody used to detect any quail-positive mesenchyme (Fig. 5I, *n* = 4/4). Only a small number of cells were grafted along with the RPE, and these remained closely associated with it. Hence, these do not explain the large number of ectopic cartilage cells surrounding the graft. Additionally, as these quail mesenchyme cells are found so close to the RPE, they would contribute to, but not totally account for, the non-cartilage tissue that is found immediately adjacent to and surrounding each graft (* in Fig. 5E).

### Mice do not have scleral cartilage but do express early cartilage markers

Unlike the majority of vertebrates, mammals do not have a cartilage layer within the sclera (Franz-Odendaal and Vickaryous, 2006). Using Alcian blue as a marker for cartilage we confirmed that this is the case in the mouse embryo (Figs. 6A–C). Whole-mount E15.5 mouse heads were stained with Alcian blue and Alizarin red. Other cranial cartilages are well differentiated and eye development is roughly equivalent to the HH stage 36 (E10) chick (Fig. 6A). Sections of the head at this stage show a faint blue staining adjacent to the optic cup, but no definitive cartilage within the sclera (Figs. 6B, C). This was also examined in E17.5 mouse heads and definitive scleral cartilage was also not present at this stage (data not shown).

To investigate whether the cartilage differentiation pathway is never initiated in the mouse, or whether it starts to develop and is then halted, early cartilage markers were analysed using in situ hybridisation. Surprisingly, the early cartilage marker *Cart1* is very strongly expressed surrounding the optic cup at E10.5 (Figs. 6D, D′, I) and, as seen in chick, this expression is stronger nasally. Furthermore, *Sox9* is also expressed adjacent to the mouse RPE at E11.5 (Figs. 6E, E′, I); however, this is at a lower level than seen in chick and can only be identified clearly in section (Fig. 6E′). The expression within the sclera of the late cartilage marker *Aggrecan* is weak and patchy at E12.5 however this cannot be detected at later stages (Figs. 6F, F′, I). It is important to note that expression seen around the eye in whole mounts (arrowhead in Figs. 6E, F) is found within the developing eyelids not the sclera. Thus, the mouse sclera does express early cartilage markers robustly but later markers are weak or absent.

The ability of mouse peri-ocular mesenchyme to form mature cartilage *in vitro* was established by performing micromass cultures of this tissue taken from E12.5 embryos (Fig. 6G). When cultured for 4 days, all 12 cultures (16 embryos, 2 litters) produced cartilage nodules with an average of 3 nodules per culture (Fig. 6H), suggesting this tissue has the ability to form mature cartilage but is unable to do so *in vivo*. The fact that the early cartilage markers are expressed in the mouse sclera suggests that the cartilage pathway is activated as in other vertebrates. However, it only progresses in a few cells that never fully mature and produce matrix, highlighting a key difference between bird and mammalian scleral development.

The difference between scleral development in chick and mouse could be due to differences in the RPE, with mouse RPE having the inability to induce cartilage en mass. To test whether mouse RPE can induce cartilage in competent mesenchyme, mouse RPE (E10.5–11.5) was grafted into HH stages 18–19 (E3) chick cranial mesenchyme as above. Unfortunately, only 4/65 embryos grafted with E10.5 RPE and 20/75 grafted with E11.5 RPE survived. Of these no grafts were seen, and we were therefore unable to test this theory. It could be that mouse RPE is dispersed, extruded or unable to grow large enough to be detected.

### Investigating signalling pathways involved in scleral cartilage development

**Fig. 7:**
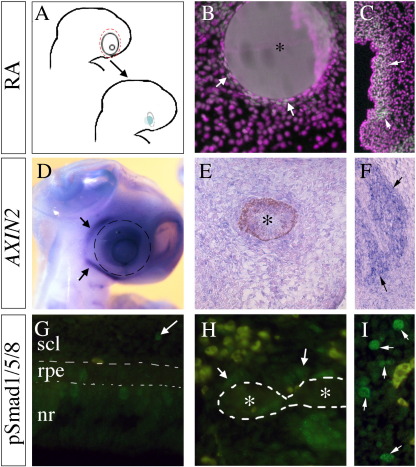
Investigation of signalling pathways involved with scleral cartilage development (A) Schematic illustrating removal of the optic cup from an HH stages 12–14 (E2) embryo and its replacement with an RA-soaked bead. (B, C) Pseudo-coloured immunostaining against SOX9 (white) with DAPI labelling of nuclei (purple), showing low expression in cells (arrows) surrounding the RA bead (*; B). Strong SOX9 expression can be seen in other regions of the head (arrows in C). (D) In situ hybridisation of a whole-mount embryo showing expression of *AXIN2* at HH stage 20 (E4), expression is seen surrounding the temporal region of the eye outside the region of the developing scleral cartilage (dashed ring). (E, F) In situ hybridisation of a sectioned embryo where the optic cup was removed at HH stages 12–14 (E2), replaced with HH stage 19 (E3) RPE and collected 3 days later. There is no upregulation of expression of *AXIN2* in the region surrounding the RPE graft (*; E), compared to the strong expression seen elsewhere (arrows in F). (G–I) Immunostaining against PHOSPHO-SMAD 1/5/8 (green) with auto-fluorescing blood cells (yellow). (G) Staining is seen in cells of the neural retina (nr), RPE and sclera surrounding the eye of an HH stages 25–27 (E5). A small number of positive cells are within the sclera (arrow). A few positive cells (arrows) can be seen around the graft (*; H), but this is far less than expression seen in other regions of the head (arrows in I).

There are a number of signalling molecules known to regulate cartilage development, many of which are known to also play a role in development of the RPE. Retinoic acid (RA) is one such molecule. To investigate the effect of retinoic acid on scleral cartilage development, the optic cup was removed at HH stages 12–14 (E2) and an RA-soaked bead inserted into the cavity (Fig. 7A). After 2 days, the embryos were collected and anti-SOX9 antibody used to detect activation of the cartilage pathway (Fig. 7B). With 0.1 mg/ml RA, a small number of mesenchymal cells immediately adjacent to the bead were positive for SOX9. However, 1 mg/ml RA did not induce this expression. This suggests that low concentrations of RA may have a role in scleral cartilage induction, but it is unlikely to be the main factor involved.

*AXIN2* is transcriptionally regulated by the Wnt pathway (Jho et al., 2002). Thus, it can act as a read-out of Wnt signalling. A number of Wnts are expressed by the RPE (Jin et al., 2002) and could be signalling to induce scleral cartilage. Using whole-mount in situ hybridisation, *AXIN2* expression was seen around the temporal regions of the eye at HH stages 20–25 (Fig. 7D, HH stage 20), but not corresponding to the developing scleral cartilage (dashed ring in Fig. 7D). This suggests that Wnts do not have a role in early scleral cartilage development. Furthermore, a HH stages 18-19 (E3) RPE graft inserted into the cavity of a HH stages 12–14 (E2) enucleated embryo and incubated for 3 days does not induce ectopic *AXIN2* expression in the mesenchyme surrounding the graft (Fig. 7E).

BMPs are another group of signalling molecules known to play a role in RPE development (Müller et al., 2007). To test whether BMPs are signalling from the RPE to induce cartilage in the surrounding neural crest-derived mesenchyme, an antibody against PHOSPHO-SMAD1/5/8 was used. At HH stages 20–27 (E4–6) there are few positive cells found within the presumptive scleral cartilage (Fig. 7G, HH stage 25). Similarly, grafting of a HH stages 18-19 (E3) RPE into the cavity of a HH stages 12–14 (E2) enucleated embryo did not result in an increase in PHOSPHO-SMAD1/5/8-positive cells in the peri-orbital mesenchyme surrounding the replacement graft (Fig. 7H). These data suggest that Wnt and Bmp are not the key molecules involved in scleral cartilage development.

## Discussion

This paper is the first in depth analysis of scleral cartilage development in the whole animal. We have found that it occurs in a series of defined developmental stages with expression of pre-commitment cartilage markers (*SOX9* and *CART1*) preceding that of the later cartilage marker *AGGRECAN* and the definitive cartilage Alcian blue stain. C*ART1* and *SOX9* are first expressed at HH stage 15 and 18 (E2.5 and E3), respectively, *AGGRECAN* is first expressed around HH stage 26 (E5) and Alcian blue staining is not seen until HH stage 34 (E8). From start to finish this process comprises of around 5.5 days. Although the order of these stages fits with previous data examining limb cartilages, the duration is markedly different, being significantly longer in the head (5.5 days vs. 3 days in the limb) (Melnick et al., 1981; Chimal-Monroy et al., 2003). This may reflect the need to delay overt cartilage formation due to rapid growth of the CNS during these stages of development.

Previous papers had examined the ability of eye structures to direct cartilage development in mesenchyme *in vitro* (CAM grafts) and suggested that the RPE played a role in this process (Newsome, 1972; Stewart and McCallion, 1975; Smith and Thorogood, 1983). However, the analysis was limited to non-specific histological staining and the presumptive cartilage formed as rods or pellets immediately adjacent to the RPE, without any evidence of a choroid. This is unlike normal scleral cartilage *in vivo*, which is a sheet of cartilage that surrounds the RPE but is separated from it by a choroid layer. We have extended these previous analyses to the *in vivo* system and show that the absence of the optic cup results in loss of scleral cartilage. Grafting of RPE into scleral mesenchyme can induce cartilage formation and in the absence of an eye, an RPE graft alone can induce the expression of cartilage markers such as *SOX9.* Also, grafting of RPE but not retina induces scleral cartilage in neural crest-derived mesenchyme that normally only forms bone. Importantly, all of these grafts adopted a similar structure to that in the normal eye, namely an RPE core, surrounded by a cartilage sheet. Interestingly, we found that these grafts also possessed a distinct non-cartilage layer, the presumptive choroid, similar to that found in the normal eye. This supports previous data that highlight the importance of RPE in choroid development (Zhao and Overbeek, 2001) and suggests that the chick RPE not only directs scleral cartilage formation but also influences choroid formation. This would imply that a diffusible factor is involved in scleral cartilage induction, data which is contradictory to that found by Smith and Thorogood (1983). In our quail grafting experiments, small numbers of mesenchymal cells were carried with the RPE graft and contributed to the presumptive choroid region and it could be suggested that it is these cells and not the RPE graft that carries the signal for cartilage induction. It has been shown that culturing early peri-ocular mesenchyme in CAM culture, which would also contain these presumptive choroid cells, does not result in the induction of cartilage (Newsome, 1972). However, we do not rule out later reciprocal signalling between these tissues being required for normal development.

Our *in vivo* analysis has also allowed us to ask whether the RPE has an inductive or merely a maintenance role in cartilage development. We have shown that the earliest molecular markers of developing cartilage are not switched on after removal of the optic cup, indicating that it induces the expression of these genes and that grafts of the RPE can ectopically induce them. Similarly we can say that it does not simply play a maintenance role in cell survival, as we did not see dramatic cell death of the mesenchyme surrounding it. Thus, the RPE has an inductive role in cartilage development and differentiation *in vivo*.

In order to identify the nature of the signal that emanates from the RPE, we have examined a number of signalling pathways that play a role in both cartilage and RPE development. Retinoic acid signalling is known to play a role in eye development (reviewed by Cvekl and Wang, 2009) and it has been demonstrated that the RPE synthesises RA throughout development (Mey et al., 2001; Prabhudesai et al., 2005). RA signalling plays a role in skeletal patterning and it has been shown that application of RA to the limb or upper beak (the latter in conjunction with Noggin) causes duplication of skeletal elements (Tickle et al., 1982; Lee et al., 2001). However, analysis of the effect of RA signalling on limb mesenchymal cultures shows that RA inhibits *SOX9* expression and prevents cartilage differentiation (Weston et al., 2002). In contrast, we saw some induction of SOX9 expression in mesenchyme immediately adjacent to the bead using low concentrations of RA, indicating that it may indeed play a role in the induction of cartilage by the RPE. However, the low level of this induction indicates that RA signalling is unlikely to be the primary mechanism of induction.

The canonical Wnt signalling pathway has recently been identified as a key player in RPE development (Westenskow et al., 2009; Fujimura et al., 2009). Similarly the role of Wnt signalling in cartilage development has been well studied and indicates that Wnt signalling can both inhibit and promote cartilage development (Yano et al., 2005; Rudnicki and Brown, 1997; Church et al., 2002, reviewed in Chun et al., 2008). Analysis of the expression of Axin2, which is transcriptionally induced by Wnt signalling, indicates while there is active signalling in the peri-ocular mesenchyme, it is not in the cartilage anlagen. This is consistent with the findings of many studies indicating that Wnt signalling inhibits early cartilage development. In keeping with this, RPE grafts were unable to induce Axin2 signalling in adjacent mesenchyme.

BMP signalling is known to be critical for RPE development and is found expressed in the RPE at many stages of development (Müller et al., 2007). BMP signalling also regulates every phase of cartilage development from condensation to hypertrophy (reviewed by Wu et al., 2007). Analysis of pSMAD 1/5/8 expression demonstrated that it was not present at high levels in the sclera mesenchyme during early development. RPE grafts did not induce high levels of pSMAD1/5/8 in adjacent mesenchyme, indicating that, similar to Wnt, BMP signalling through the SMAD pathway is unlikely to be playing a major role in induction of sclera cartilage by the RPE.

It must be noted that these experiments have not addressed the role of RPE in the development of the fibrous sclera. It has been shown that proliferation of chick fibrous scleral cells in culture requires co-culture with RPE (Seko et al., 1994), implying a diffusible RPE signal influences scleral development. The transcription factor Pitx2 is required for adoption of the sclera fate (Evans and Gage, 2005) and it has recently been shown that RA from the optic cup induces Pitx2 expression in the anterior chamber of the eye (Gage and Zacharias, 2009; Kumar and Duester, 2010). Thus, it is possible that RA signalling from the RPE weakly induces sclera cartilage markers (*SOX9*) but strongly induces sclera markers (*PITX2*) along a gradient, thus directing fate choices within the neural crest population that forms both of these cell types.

While, scleral cartilage can be seen surrounding, and at a short distance, from the RPE, it is not continuous around the whole eye, as there is a gap for the optic nerve to exit the eye and there is no cartilage covering the iris or cornea. This suggests inhibitory molecules may play a role in the formation of correct cartilage shape, and it would be interesting to look at expression of known cartilage inhibitory molecules in these regions. Interestingly, in some fish, the shape of scleral cartilage is different (Franz-Odendaal and Hall, 2006; Franz-Odendaal and Vickaryous, 2006), it forms a ring around the eye rather than a cup, this could be due to differing expression patterns of inhibitory molecules or to differences in the inductive potential of the RPE.

Our analysis shows that scleral cartilage differentiation does not occur concurrently around the eye but is initiated anteriorly, adjacent to the peripheral retina and then progresses posteriorly. In contrast, the retina and RPE mature in the opposite direction with terminal differentiation of retinal and RPE cells occurring first in central regions (McCabe et al., 1999). However, the pigmentation of the RPE is in an identical pattern to the cartilage differentiation, suggesting an intriguing link between the development of pigmentation and cartilage.

Mammals are a vertebrate group that does not possess a scleral cartilage. However, we have shown in mice that this structure does start to form but that this developmental process is arrested. In addition we have shown that mouse scleral mesenchyme is able to make cartilage *in vitro*, albeit at relatively low levels. This might suggest that there are small populations of cells within the mouse sclera that retain their cartilage potential and act as a stem cell population. Intriguingly, there are pathological instances where scleral cartilage and bone have been reported in human sclera (Traboulsi et al., 1999; Pecorella et al., 2006) and there is also a link between diseases of cartilage and the sclera. Both the sclera and joint cartilage are common targets in rheumatoid arthritis (Seko et al., 2008), an autoimmune disease that predominantly attacks the joints causing inflammation and progressive tissue destruction, but which also causes chronic scleral inflammation known as refractory scleritis. Microarray data on cultured human infant scleral cells showed similar profile to cartilage derived cells, and culture studies of this tissue lead to the expression of cartilage-associated genes (Seko et al., 2008). These data suggest that human sclera may retain the ability to form cartilage as in other vertebrates, and be affected by cartilage diseases. Our data suggest that during mouse development scleral mesenchyme does undergo at least the earliest phases of cartilage development and that stem cells with the ability to form cartilage may remain in this tissue. In certain pathological conditions this may be further elaborated in the adult. In light of this it is also interesting to note that a high proportion of aging rats have cartilage formation in the sclera (O'Steen and Brodish, 1990). Thus, this may not only be a mechanism in pathological disease but may also occur due to aging.

In conclusion, we have shown the stages of scleral cartilage development in the chick and found that this cartilage develops over an extended period of time compared to limb cartilage. We highlight the importance the optic cup in initiation of scleral cartilage development and prove *in vivo* that the RPE is the tissue that induces this cartilage. Furthermore, we found that even though mammals do not have scleral cartilage, cartilage development is initiated in mouse.
